# Statistical Optimization of Fibrinolytic Enzyme Production Using Agroresidues by *Bacillus cereus* IND1 and Its Thrombolytic Activity *In Vitro*

**DOI:** 10.1155/2014/725064

**Published:** 2014-06-09

**Authors:** Ponnuswamy Vijayaraghavan, Samuel Gnana Prakash Vincent

**Affiliations:** International Centre for Nanobiotechnology, Centre for Marine Science and Technology, Manonmaniam Sundaranar University, Rajakkamangalam, Kanyakumari District, Tamil Nadu 629 502, India

## Abstract

A potent fibrinolytic enzyme-producing *Bacillus cereus* IND1 was isolated from the Indian food, rice. Solid-state fermentation was carried out using agroresidues for the production of fibrinolytic enzyme. Among the substrates, wheat bran supported more enzyme production and has been used for the optimized enzyme production by statistical approach. Two-level full-factorial design demonstrated that moisture, supplementation of beef extract, and sodium dihydrogen phosphate have significantly influenced enzyme production (*P* < 0.05). A central composite design resulted in the production of 3699 U/mL of enzyme in the presence of 0.3% (w/w) beef extract and 0.05% (w/w) sodium dihydrogen phosphate, at 100% (v/w) moisture after 72 h of fermentation. The enzyme production increased fourfold compared to the original medium. This enzyme was purified to homogeneity by ammonium sulfate precipitation, diethylaminoethyl-cellulose ion-exchange chromatography, Sephadex G-75 gel filtration chromatography, and casein-agarose affinity chromatography and had an apparent molecular mass of 29.5 kDa. The optimum pH and temperature for the activity of fibrinolytic enzyme were found to be 8.0 and 60°C, respectively. This enzyme was highly stable at wide pH range (7.0–9.0) and showed 27% ± 6% enzyme activity after initial denaturation at 60°C for 1 h. *In vitro* assays revealed that the enzyme could activate plasminogen and significantly degraded the fibrin net of blood clot, which suggests its potential as an effective thrombolytic agent.

## 1. Introduction

Cardiovascular diseases (CVDs) including acute myocardial infarction, ischemic heart disease, peripheral vascular disease, high blood pressure, and stroke are the leading causes of death worldwide [1]. According to the World Health Organization, it is estimated that 17.3 million people died from CVDs in 2008 and that it would be more than 23 million people by 2030 [2]. A variety of thrombolytic agents such as tissue plasminogen activator (t-PA), urokinase plasminogen activator (u-PA), and streptokinase were used to treat CVDs. In the cases of t-PA and u-PA, they are generally safe but very expensive. In contrast, bacterial streptokinase is a cheap thrombolytic agent but it is life-threatening [3]. Hence, the search for a novel fibrinolytic agent to treat CVDs continues. Nattokinase, a fibrinolytic enzyme isolated from* Bacillus natto*, had potent thrombolytic activity. These bacterial fibrinolytic enzymes are generally safe, and the oral administration of the nattokinase enzyme could increase the fibrinolytic activity in human plasma [4]. Fibrinolytic enzymes can be found in a variety of foods, such as Japanese* natto*,* tofuyo*, Korean* cheonggukjang* soy sauce, edible honey mushroom [1], Chinese* douche* [5], Indonesian* tempeh* [6], Taiwanese fermented red bean [7], Japanese shiokara [8], and Asian fermented shrimp paste [9]. These fibrinolytic enzymes possess three antithrombotic activities, which included the conversion of plasminogen to plasmin, the activation of t-PA, and finally the degradation of fibrin by fibrinolytic activity of plasmin in conjunction with nattokinase [10].

Solid-state fermentation (SSF) has emerged as a potential technology for the production of pharmaceutically significant products. Many agroindustrial residues were used for the production of these products using SSF. The agroindustrial residues such as pigeon pea, green gram husk, potato peel, and wheat bran were widely used for the production of proteases [11–14]. Utilization of these agroindustrial residues as substrates in SSF processes provides an alternate avenue and value addition. Product's yield is mostly higher using SSF when compared with submerged fermentation. Selection of an appropriate substrate is another key aspect of SSF [14]. Although the fibrinolytic enzyme production was found to be high while using SSF, no much work on the use of SSF and the statistical approach of optimization of fibrinolytic enzyme production has been carried out. SSF was employed for the production of fibrinolytic enzymes with few solid substrates (rice chaff and* Fusarium oxysporum* [15] and* Bacillus firmus* NA-1 and soybean grits [16]). Response surface methodology (RSM) has been used widely for the production of various enzymes, including polygalacturonase [17], arginine deiminase [18], *α*-amylase [19], acid protease [20], and *β*-galactosidase [21]. However, there are few studies on the optimization of medium components for microbes to produce fibrinolytic enzymes via two-level full-factorial design and RSM [22].

The aim of this work was to optimize the fermentation medium by RSM to increase fibrinolytic enzyme production in SSF. The optimization procedure included the following: a two-level full-factorial design, a central composite design (CCD), and an RSM. The extracted fibrinolytic enzyme was purified by chromatographic methods and was used to study its blood clot lytic properties* in vitro*.

## 2. Materials and Methods

### 2.1. Screening of Fibrinolytic Enzyme-Producing Organisms from the Indian Rice

Approximately 1.0 g of fermented rice was transferred to an Erlenmeyer flask (250 mL) with 50 mL of sterile double-distilled water, shaken vigorously for 10 min, and 1 mL of this solution was resuspended in sterile double-distilled water and aliquots were then spread on nutrient agar plates composed of the following (g/L): peptic digest of animal tissue, 5.0; beef extract, 1.5; yeast extract, 1.5; sodium chloride, 5.0; and 10 skimmed milk (pH 7.0). Colonies, indicated by clear zones corresponding to protease activity, were selected and cultured in SSF using wheat bran as the substrate. SSF was performed by inoculating 10% (v/w) precultured* Bacillus cereus* and incubated at 37°C for 72 h. The pH of the fermenting medium was maintained as 8.0 using Tris buffer (0.1 M) and 100% (v/w) moisture. Fibrinolytic activity of the crude enzyme was tested in a fibrin plate composed of 0.1 M sodium phosphate buffer (pH 7.4), 1% (w/v) agarose, 1.2% (v/v) fibrinogen, and thrombin (100 NIH units/mL) [23]. The fibrin plate was allowed to stand for 1 h at room temperature to form a fibrin clot layer. About 10 *μ*L of crude enzyme was dropped into holes and incubated at 37°C for 5 h. The fibrinolytic enzyme exhibited a clear zone of degradation of fibrin around the well, thus indicating its fibrinolytic activity.

### 2.2. Identification of the Fibrinolytic Enzyme-Secreting Organism

The isolated strain IND1 with highest activity was identified on the basis of the biochemical properties, the phenotypical characteristics [24], and the 16S rRNA gene sequencing. The genomic DNA was extracted from the cells of an 18-h cultured IND1 strain by using a QIAGEN DNA purification kit (Germany) according to the manufacturer's instructions. The 16S rRNA gene of the isolate was amplified by polymerase chain reaction (PCR) using the upstream primer P1: 5′-AGAGTTTGATCMTGGCTAG-3′ and the downstream primer P2: 5′-ACGGGCGG TGTGTRC-3′ (Sigma-Aldrich). Amplification of DNA was carried out using the research gradient Peltier Thermal cycler machine PTC-225 and DNA polymerase (Sigma) under the following conditions: denaturation at 95°C for 3 min followed by 30 cycles at 95°C for 1 min, 55°C for 30 s, and 72°C for 1 min and 50 s. The amplified product was sequenced at Scigenome Laboratories, India. Sequence comparison with databases was performed using BLAST through the NCBI server [25]. The isolate IND1 was identified as* B. cereus* IND1. The 910 bp sequences were submitted to the GenBank database, and an accession number was assigned to those sequences. The GenBank accession number of the sequence reported in this paper is KF250417.

### 2.3. Evaluation of Agroindustrial Residues for Fibrinolytic Enzyme Production

The substrates such as banana peel, tapioca peel, rice bran, wheat bran, and green gram husk were collected locally and dried for several days and powdered. About 2.0 g of substrates was taken in an Erlenmeyer flask, and the moisture content was maintained as 100% level. The contents were mixed thoroughly and inoculated with 0.2 mL of 18 h grown culture (OD 600 nm = 1.08). To the fermented medium, 20 mL of double-distilled water was added and placed in an orbital shaker at 150 rpm for 30 min. After this, it was centrifuged at 10,000 ×g for 10 min, and the supernatant was used as the crude enzyme.

### 2.4. Assay of Fibrinolytic Activity

Fibrinolytic activity of the sample was measured by the hydrolysis of fibrin [26]. The reaction mixture contained 2.5 mL of 1.2% (w/v) fibrin (pH 7.8), 2.5 mL of 0.1 M Tris-HCl buffer (containing 0.01 M CaCl_2_, pH 7.8), and 0.05 mL of enzyme solution. After 30 min at 37°C, the reaction was stopped by adding 5 mL of 0.11 M trichloroacetic acid containing 0.22 M sodium acetate and 0.33 M acetic acid. The reaction mixture was centrifuged at 10,000 ×g for 10 min, and the absorbency of the sample was read at 275 nm against a sample blank. A fibrinolytic unit was defined as the amount of enzyme that gave an increase in absorbency at 275 nm equivalent to 1 *μ*g of tyrosine/min at 37°C. The total protein content determination was performed as described by Lowry et al. [27].

### 2.5. Evaluation of Significant Factors with 2^5^ Factorial Design

The main factors that significantly influence fibrinolytic enzyme production were screened using 2^5^ factorial design and the RSM. With the results obtained from the one-at-a-time strategy, three nutrients were selected for further optimization. Important nutrient factors such as maltose, beef extract, and NaH_2_PO_4_ were added to the solid substrate according to 2^5^ full-factorial design. From an SSF point of view, moisture content is one of the significant factors. Hence, the optimum moisture content and pH were evaluated with respect to the production of the fibrinolytic enzyme. Based on the two-level full-factorial design, each factor was examined at two levels: −1 for low level and +1 for high level.  Table 1 lists the variables and levels (high and low) in detail. Fibrinolytic activity assay was carried out in duplicates, and the average value was taken as response *Y* (Table 2). Analysis of variance (ANOVA) was used to estimate the statistical parameters, and the values of “Prob >*F*” < 0.05 indicate that the model terms are significant (Table 3). Statistical software (Design-Expert 8.0.7.1; StatEase Inc., Minneapolis, Minnesota) was used to design and analyze the experiment. The significant main effect can be calculated using (1) after neglecting the insignificant main effects.

Consider the following

(1) $$\begin{array}{c} Y=\alpha_{0}+\;\overset{}{\underset{i}{\sum }}\alpha_{i}x_{i}+\overset{}{\underset{ij}{\sum }}\alpha_{ij}x_{i}x_{j}+\overset{}{\underset{ijk}{\sum }}\alpha_{ijk}x_{i}x_{j}x_{k}, \end{array}$$

where *α*_*ij*_ and *α*_*ijk*_ are the *ij*th and *ij* *k*th interaction coefficients, respectively, *α*_*i*_ is the *i*th linear coefficient, and *α*_0_ is an intercept.

### 2.6. Optimization of Enzyme Production by RSM

RSM and the CCD were employed to optimize the most significant factors (moisture, beef extract, and NaH_2_PO_4_) regarding the fibrinolytic enzyme production. Each of the variables used was analyzed at five coded levels (−*α*, −1, 0, +1, +*α*) as listed in Table 4. According to the Design-Expert 8.0.7.1, a CCD design of three factors consists of 20 runs (eight factorial, six axial, and six center points). Central point of the CCD is the actual level of variables designed on the basis of initial experiments (one factor at a time and the 2^5^ factorial design). About 2.0 g of wheat bran was taken in an Erlenmeyer flask and mixed with a predetermined quantity of Tris buffer (0.1 M, pH 8.0), and a calculated amount of beef extract and NaH_2_PO_4_ was also added to the flask. The substrate and nutrients were mixed carefully and sterilized at 121°C for 20 min. All the Erlenmeyer flasks were inoculated with a 10% (v/w) inoculum and incubated at 37°C for 72 h. Twenty milliliters of sterilized double-distilled water was added to extract the fibrinolytic enzyme unless otherwise stated. The enzyme activity assay was carried out in duplicates, and the average of these experimental values was taken as response *Y* (Table 5). Values of “Prob >*F*” < 0.05 indicate that the model terms are significant. In this model, the *P* value was <0.05; hence this model was significant (Table 6). The experimental results of the CCD were fitted with a second-order polynomial equation as shown in (2).

Consider the following:

(2) Y=α0+α1A+α2B+α3C+α1α2AB+α1α3AC+α2α3BC+α1α1A2+α2α2B2+α3α3C2,

where *Y* is the fibrinolytic activity (units/mL); *A* is the coded value of moisture; *B* is the coded value of the beef extract; *C* is the coded value of NaH_2_PO_4_; *α*_1_, *α*_2_, and *α*_3_ are the linear coefficients; *α*_1_ *α*_2_, *α*_1_ *α*_3_, and *α*_2_ *α*_3_ are the interactive coefficients; and *α*_1_ *α*_1_, *α*_2_ *α*_2_, and *α*_3_ *α*_3_ are the quadratic coefficients.

Response surface graphs were plotted to determine the optimum fibrinolytic enzyme production. The fitted polynomial equation was expressed as three-dimensional (3D) surface plots to visualize the relation between responses and the experimental levels of each factor used in the design. Validation of the model was performed under the conditions predicted by the model. SSF was carried out, and fibrinolytic activity was assayed as described earlier.

### 2.7. Purification of Fibrinolytic Enzyme

The crude extract was precipitated by the addition of solid ammonium sulfate at 30%–70% saturation. The precipitate was allowed to form at 4°C overnight and was collected by centrifugation at 10,000 ×g in a refrigerated centrifuge for 15 min. The precipitate was dissolved in 5 mL of buffer A (0.025 M sodium phosphate buffer, pH 7.0). It was dialysed against buffer A overnight and loaded on DEAE-cellulose (Merck, Bangalore) column, which was preequilibrated with buffer A. The column was washed with buffer A to remove all unbound proteins, and a linear gradient of 0–0.75 M NaCl-added buffer A was used to elute the bound proteins. Fractions exhibiting fibrinolytic activity were pooled and concentrated with ammonium sulfate. The precipitate was collected by centrifugation at 10,000 ×g in a refrigerated centrifuge at 4°C for 15 min, dissolved in buffer A, and dialysed against the same buffer overnight. This sample was loaded on Sephadex G-75 (Amersham Biosciences, Sweden) gel filtration column (0.7 × 45 cm) which was preequilibrated with buffer A. The eluate was monitored for protein concentration at 280 nm and was assayed for fibrinolytic activity. Fractions with high fibrinolytic enzyme activity were pooled and loaded on casein-agarose affinity column (Sigma) and washed with buffer A. The fibrinolytic enzyme was eluted with buffer A containing 0.1, 0.2, 0.3, 0.4, 0.5, 0.7, and 0.8 M NaCl. All fractions were subjected to fibrinolytic activity assay. The purified enzyme was stored at −20°C and used for characterization studies.

### 2.8. Sodium Dodecyl Sulfate-Polyacrylamide Gel Electrophoresis and Molecular Mass Determination

Sodium dodecyl sulfate-polyacrylamide gel electrophoresis (SDS-PAGE, 12%) was performed to determine the molecular mass of the fibrinolytic enzyme following the methods of Laemmli [28]. The molecular weight of the fibrinolytic enzyme was estimated with phosphorylase b (97.4 kDa), bovine serum albumin (66 kDa), ovalbumin (43 kDa), carbonic anhydrase (29 kDa), soybean trypsin inhibitor (20.1 kDa), and lysozyme (14.3 kDa) markers.

### 2.9. Properties of Fibrinolytic Enzyme

The optimum pH for the activity of an enzyme was determined using the following buffers (0.1 M): citrate buffer (pH 3.0 and 4.0), succinate buffer (pH 5.0), sodium phosphate buffer (pH 6.0 and 7.0), Tris buffer (pH 8.0), and glycine-NaOH buffer (pH 9.0 and 10.0). The stability of fibrinolytic enzyme activity in response to pH was evaluated by incubating the enzyme with the above buffers at 37°C for 1 h prior to incubation with substrate. The effect of temperature on enzyme activity was determined by performing the reactions at various temperatures: 30, 40, 50, 60, and 70°C. To determine the thermal stability, the fibrinolytic enzyme was incubated (without substrate at various temperatures, 30–70°C) for 1 h. Enzyme activity was assayed as described earlier. To study the effect of divalent ions on enzyme activity, the enzyme sample was incubated with various divalent ions, namely, Ca^2+^, Co^2+^, Cu^2+^, Mg^2+^, Mn^2+^, Hg^2+^, Fe^2+^, and Zn^2+^. The enzyme activity was determined as described earlier.

### 2.10. Analysis of Fibrinolysis on Plasminogen-Rich and Plasminogen-Free Fibrin Plates

The plasminogen-rich and plasminogen-free plates were prepared to evaluate the efficacy of fibrinolytic enzyme on plasminogen and direct fibrin clot lysis. The fibrin plate was heated at 80°C for 30 min to inactivate plasminogen present with the commercially available fibrinogen. Twenty microliters of fibrinolytic enzyme was placed on the plasminogen-free and plasminogen-rich plates and incubated at room temperature for 4 h. The enzyme exhibited a clear zone of degradation of fibrin on plasminogen-rich plate and plasminogen-free plate was observed.

### 2.11. Blood Clot Lysis of Fibrinolytic Enzymes on Human Blood

The clot lytic effect of fibrinolytic enzyme was studied with an artificial clot* in vitro*. The blood was collected from healthy male volunteer with written informed consents. Artificial blood clot was made by spontaneous coagulation in the centrifuge vials. Then, different doses (100, 200, and 300 units) of the fibrinolytic enzyme were added. Streptokinase (250 units) was used as the positive control; buffered saline solution was used as the negative control. These were incubated at room temperature for 60 min and analyzed.

## 3. Results and Discussion

### 3.1. Screening of a Potent Fibrinolytic Enzyme-Producing Strain

The bacterium* B. cereus* IND1 displayed more activity on skimmed milk agar plates and fibrin plates. It produced approximately an 11 mm zone on the fibrin plate, which was higher than the other isolates. The isolated strain was Gram positive, rod shaped, catalase positive, and oxidase positive and had tested negative for indole formation, citrate utilization, and the hydrolysis of urea. It was able to hydrolyze starch and casein and also tested negative for gelatin hydrolysis.

### 3.2. Utilization of Agroresidues for the Production of Fibrinolytic Enzyme

**Figure 1:**
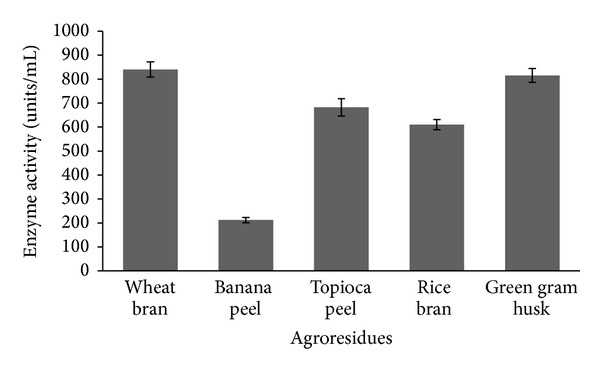
Screening of different agroresidues for the production of fibrinolytic enzyme.

The results showed that fibrinolytic enzyme production by* B. cereus* IND1 varied with the type of substrates. Enzyme production was found to be high in the wheat bran medium (840 ± 32 units/mL; Figure 1). This organism effectively utilized wheat bran for its growth and fibrinolytic enzyme production. The selection of suitable agroresidues for enzyme production in a SSF process depends on several factors including cost and availability [14].

### 3.3. Evaluation of Medium Components Affecting Fibrinolytic Enzyme Production Using RSM and 2^5^ Factorial Design

The 2^5^ factorial experimental design proved to be a valuable tool for the evaluation of the main effects of fibrinolytic enzyme production. The results of the 2^5^ factorial design have been listed in Table 2. From Table 2, the fibrinolytic activity obtained during production using the 2^5^ factorial design varied between 274 and 3267 units/mL. ANOVA was performed to verify the validity of the models, and the results have been listed in Table 3. Based on ANOVA, the “*F*-value” for the overall regression model (10.42) is significant at the 5% level. There is only a 0.80% chance that a “model *F*-value” this large could occur due to noise. In this model, *B*, *C*, *E*, *AC*, *BC*, *CD*, *ABD*, *ADE*, *BDE*, *CD* *E*, *A* *BC* *E*, and *BC* *D* *E* are significant. Maltose significantly increased the fibrinolytic enzyme production. These results were in accordance with reported fibrinolytic enzyme production in the presence of maltose for* Bacillus natto* [22]. The medium pH and the NaH_2_PO_4_ concentration affected the fibrinolytic enzyme production significantly. Enzyme production was greatly affected by moisture (*P* < 0.01), beef extract (*P* < 0.01), and NaH_2_PO_4_ (*P* < 0.05) concentrations with the medium. The equation in terms of the coded factors is given as follows:

(3) Enzyme  activity=+1649.06−111.94B+159.06C+202.50E+161.31BC+88.13BE−42.25CE+101.88BCE.

On the basis of calculated *t* values, the moisture content, beef extract, and NaH_2_PO_4_ were selected for further optimization using RSM.

### 3.4. Response Surface Methodology

RSM is a powerful technique for testing multiple process variables, because fewer experimental trials are required when compared with the study of one variable at a time. Also, interactions between variables can be identified and quantified by this technique [29]. The significant factors (moisture, beef extract, and NaH_2_PO_4_) were investigated further using CCD and RSM. The CCD model helps to study the interactions between the various variables, and RSM helps to explore the optimum concentrations of each of the variables. RSM had been successfully used for the enhancement of fibrinolytic enzyme production by the* Bacillus natto* [22],* Bacillus subtilis* [30], and* Bacillus* sp. strain AS-S20-1 [31].

The second-order polynomial model was used to correct the independent variables with fibrinolytic activity. The highest activity of the fibrinolytic protease that was observed was at 3699 units/mL at run 9 (Table 5). The model *F*-value of 9.76 implied that the model was significant (Table 6). There is only a 0.07% chance that a “model *F*-value” of this magnitude could occur due to noise. The data obtained best fitted into a quadratic model. The regression analysis of the experimental design showed that the linear model terms (*A*, *B*, and *C*) and the quadratic model term (*A* ^2^) were significant. However, the interactive model terms (*AB*, *AC*, and *BC*) and quadratic model terms (*B* ^2^ and *C* ^2^) were found to be insignificant (*P* > 0.05). Yuguo et al. [32] stated that the coefficient (*R* ^2^) could be at least 0.8 for a good fit of the model. The multiple correlation coefficient (*R* ^2^) of this model is 0.89 (a value >0.75 indicates aptness of the model), which means that the model can explain 89% of the variation in the response. The adjusted coefficient (*R* ^2^) obtained for the model was 0.805. According to ANOVA, the lack of fit is insignificant, indicating that the second-order model with interaction is very adequate in approximating the response surface of the experimental design. The lack of fit's *F*-value of 3.4 implies that there is a 9.58% chance that a large lack of fit *F*-value could occur due to noise. A nonsignificant lack of fit is good. Applying multiple regression analysis, the results were fitted into a second-order polynomial (2).

**Figure 2:**
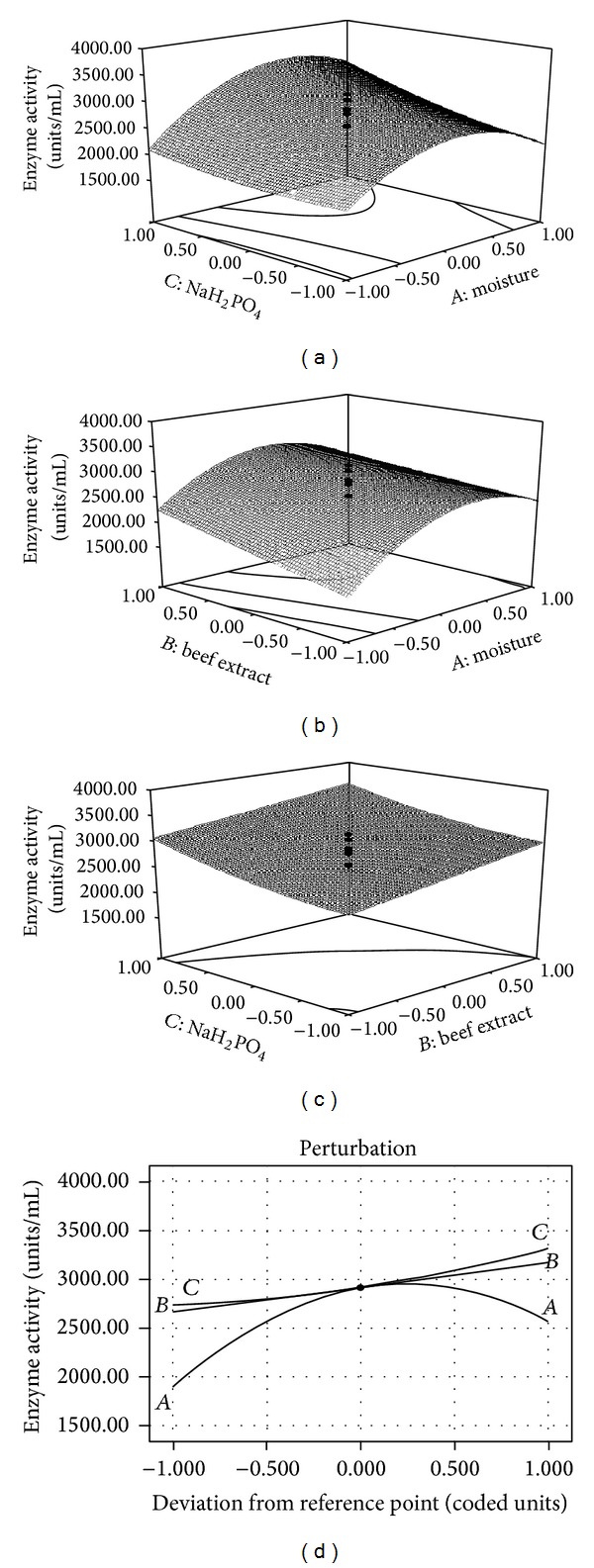
Response surface plot for fibrinolytic enzyme production by* B. cereus* IND1. (a) The interactive effects of moisture and NaH_2_PO_4_; (b) the interactive effects of moisture and beef extract; (c) the interactive effects of beef extract and NaH_2_PO_4_; (d) perturbation graph shows the effect of moisture, beef extract, and NaH_2_PO_4_ on fibrinolytic enzyme production.

Three-dimensional response surfaces were plotted on the basis of the model equation to investigate the interaction among the various variables and to determine the optimum concentration of each factor for the maximum fibrinolytic enzyme production (Figures 2(a)–2(c)). The fibrinolytic enzyme production varied significantly upon changing the initial moisture content and concentrations of the beef extract. The 3D plots depicted that there was an increased enzyme production up to 100% moisture content of the medium and then depleted thereafter. The increase of enzyme activity is attributed to a higher production of enzyme in the presence of an optimal level of moisture. This result was in agreement with Pandey et al. [14] who stated that moisture content is one of the critical factors for enzyme production in SSF. Increasing concentrations of the beef extract and NaH_2_PO_4_ resulted in the fibrinolytic enzyme production up to the optimum level. The optimal concentrations for the production of enzyme were 100% (v/w) moisture, 0.3% (w/w) beef extract, and 0.05% (w/w) NaH_2_PO_4_. The final equation in terms of coded factors for the CCD model is expressed in (2). To understand the effect of all the three factors, a perturbation plot was generated from this experiment (Figure 2(d)). A low value (14.06%) of the coefficient of variance indicates a high degree of precision and good reliability of experimental values. The RSM and the perturbation graph clearly show that sodium dihydrogen phosphate and moisture content greatly affect the production of the fibrinolytic enzyme. The “adequate precision” is a measure of signal-to-noise ratio, and a value greater than four is desirable. In this model, a ratio of 13.048 indicates an adequate signal, and this model can be used to navigate the design space. The regression equation coefficient was calculated, and the data were fitted into a second-order polynomial equation as given below:

(4) Fibrinolytic  activity (Y) =+2913.45+335.95A+249.93B  +288.80C−122.25AB+189.75AC  −11.25BC−679.04A2+4.03B2  +108.33C2,

where *A* is moisture, *B* is beef extract, and *C* is NaH_2_PO_4_.

Results revealed that the RSM-optimized medium increased fourfold of fibrinolytic enzyme production compared to the unoptimized medium. The enzyme production was comparatively higher than the earlier report of RSM on* B. subtilis* [30] and on* Bacillus* sp. strain AS-S20-1 [31].

### 3.5. Validation of the Optimized Condition

Based on the RSM result, the quadratic model predicted that the maximum production of fibrinolytic enzyme was 3705 units/mL, while the *A*, *B*, and *C* code levels were 0, 0, and 1.682 corresponding to 80% (v/w) moisture, 0.2% (w/w) beef extract, and 0.0568% sodium dihydrogen phosphate. To verify the predicted result, a validation experiment was performed in triplicates. Under optimized conditions, the observed average experimental fibrinolytic enzyme production was 3750 ± 118 units/mL, indicating that the experimental and predicted values (3670 ± 48 units/mL) were in good agreement. The fibrinolytic activity obtained from the experiments was very close to the actual response predicted by the model, which proved the validity of the model.

### 3.6. Purification of Fibrinolytic Enzyme

**Figure 3:**
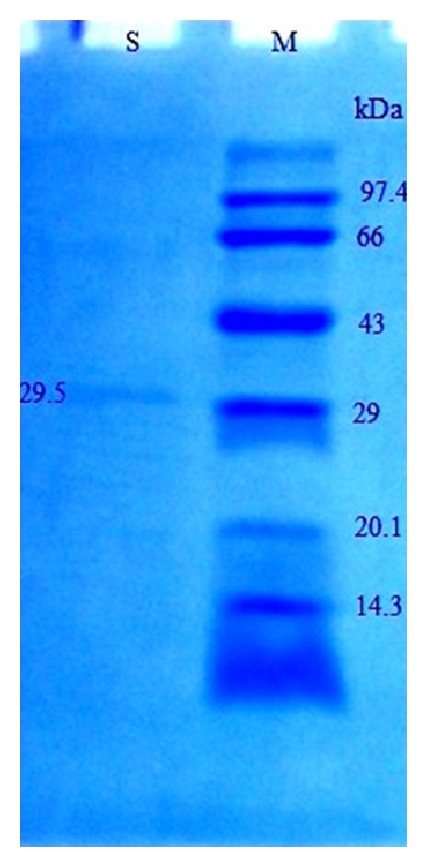
SDS-PAGE of purified fibrinolytic enzyme from* B. cereus* IND1 (S: purified enzyme after casein-agarose affinity chromatography and M: marker).

The enzyme was purified through four steps including ammonium sulfate precipitation, DEAE cellulose, gel filtration, and affinity chromatography. In the present study, the 30%–70% of saturated ammonium sulfate was found to be optimum for fibrinolytic enzyme precipitation. The recovery and purification were 4.57% and 12.8-fold, respectively, after casein-agarose affinity chromatography. The specific activity of the purified fibrinolytic enzyme was 960 units/mg protein. The purification summary is listed in Table 7. The purity of the casein-agarose affinity fractions was tested using SDS-PAGE. The SDS-PAGE shows a single band with an apparent molecular mass of 29.5 kDa, indicating the homogeneity of an enzyme (Figure 3). The* Bacillus cereus* IND1 fibrinolytic enzyme had a molecular mass comparable with that of the serine protease of* Bacillus amyloliquefaciens* DC-4 (28 kDa) [33],* Bacillus* sp. DJ-4 (29 kDa) [34], and* B. subtilis* LD-8547 (30 kDa) [35].

### 3.7. Properties of Fibrinolytic Enzymes

**Figure 4:**
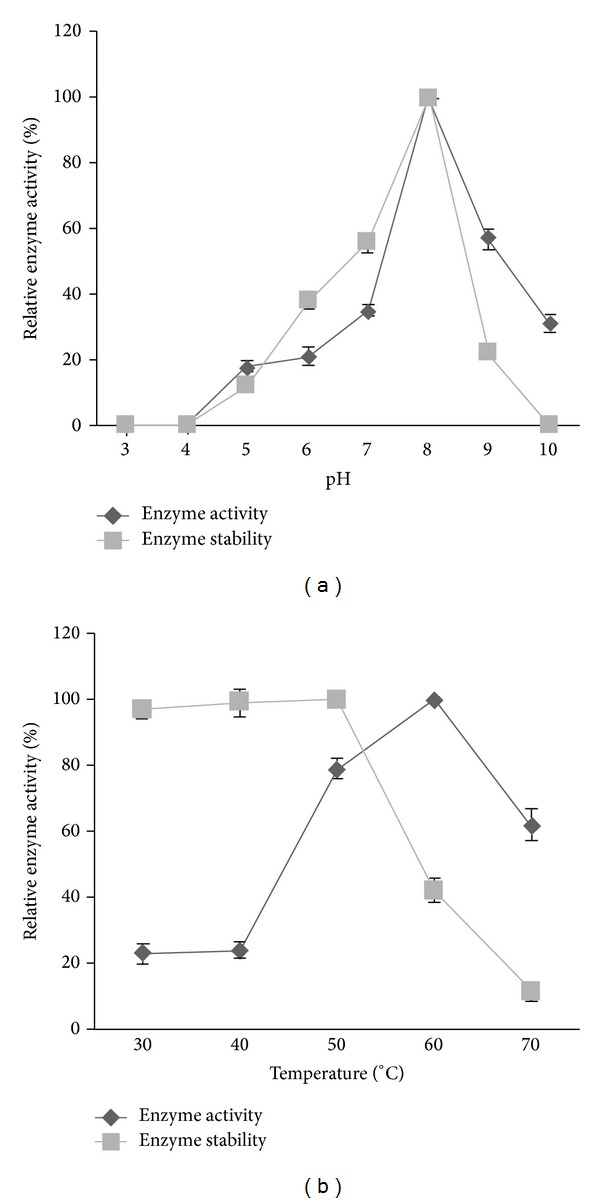
Effect of (a) pH and (b) temperature on enzyme activity and stability.

The results of optimum pH on enzyme activity and pH stability are shown in Figure 4(a). The fibrinolytic enzyme was activated at neutral and alkaline pH values, and optimal reaction for fibrinolytic enzyme was obtained at pH 8.0. The optimized pH value of the fibrinolytic enzyme was higher than many fibrinolytic enzymes, such as pH 7.0 of* Bacillus* sp. KA38 metalloprotease [36] and* B. subtilis* strain A1 nattokinase [37]. The enzyme activity was optimum at 60°C, which was higher than the* B. subtilis* natto B-12 [38]. The optimum temperature and thermostability of the enzyme are shown in Figure 4(b). As the denaturation time was increased to 1 h at 50°C, the enzyme activity decreased approximately to 40%.

### 3.8. PA Activity and Direct Clot Lytic Effect of Fibrinolytic Enzyme

**Figure 5:**
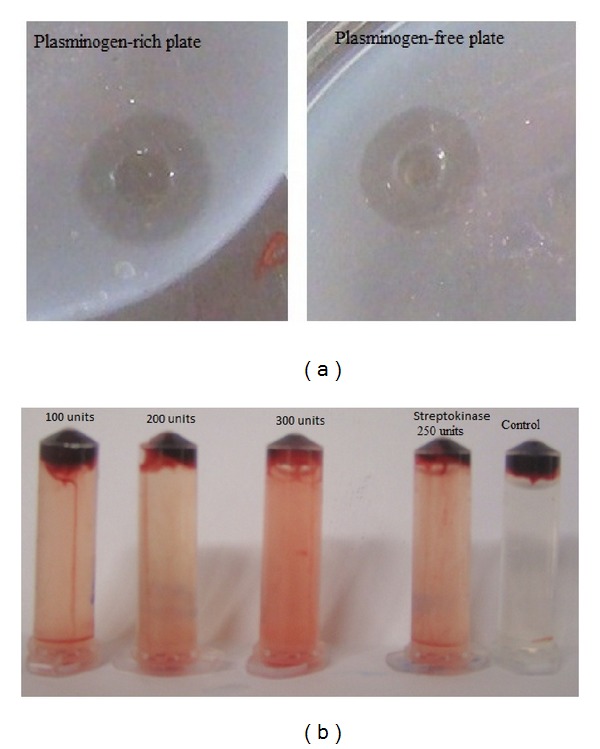
Analysis of fibrinolysis by fibrinolytic enzyme on plasminogen-rich fibrin plate and plasminogen-free plate (a).* In vitro* digestion of fibrin net of human blood clot (b). Streptokinase was used as the standard, and phosphate buffer (pH 7.4) was used as the blank.

The plasminogen-rich plate showed 1.2-fold activity compared to plasminogen-free plate (Figure 5(a)). These results demonstrate that the fibrinolytic enzyme activates plasminogen and also has plasmin-like activity. The clot lysis was studied by incubating the human blood clot with fibrinolytic enzyme. The fibrinolytic enzyme digested the fibrin net completely within 60 min of incubation at room temperature (30 ± 2°C). At higher fibrinolytic enzyme concentration (300 units), it digested fibrin net within 40 min (Figure 5(b)). In the control tube, clot degradation was not observed. These results suggested that fibrinolytic enzymes had obvious effect on dissolving blood clot, and the effect was enhanced as the concentration of fibrinolytic enzyme increased. This kind of dose-dependent clot lysis was reported with* B. subtilis* LD-8547 fibrinolytic enzymes [39]. The degradation of fibrin suggested predominant secretion of fibrinolytic protease from this strain using wheat bran as a substrate, which may have great application in the treatment of CVDs.

## 4. Conclusion 

A potent fibrinolytic enzyme was produced using agroresidues by* B. cereus* IND1. This organism effectively utilized wheat bran for the fibrinolytic enzyme production, and the process parameters were optimized by two-level full-factorial design and the RSM. This statistical approach proved to be a powerful tool for the optimization of fibrinolytic enzyme production. The optimal medium showed fourfold enzyme production compared to the unoptimized medium. Among the nutrient sources, sodium dihydrogen phosphate significantly increased enzyme production. The purified enzyme was active at higher temperatures and at wide pH ranges. It digested human blood clot* in vitro*. This study explores new sources of fibrinolytic enzymes to treat and prevent CVDs.

## Figures and Tables

**Table 1:** Independent variables and their levels for the 2^5^ factorial experimental design.

Factor	Name	Units	Coded levels	
			−1	+1
*A*	Maltose	%	0.1	0.5
*B*	NaH_2_PO_4_	%	0.01	0.1
*C*	Beef extract	%	0.05	0.25
*D*	pH		7.0	8.0
*E*	Moisture	%	60	100

**Table 2:** Results of the 2^5 ^factorial design.

Run	Factor: *A*	Factor: *B*	Factor: *C*	Factor: *D*	Factor: *E*	Response (*Y*)
1	1	−1	1	1	−1	1473
2	−1	1	−1	−1	−1	1116
3	−1	−1	1	−1	−1	2040
4	1	−1	−1	−1	1	2946
5	−1	1	−1	1	1	521
6	1	−1	−1	1	1	1200
7	−1	−1	1	−1	1	1189
8	−1	−1	−1	1	1	1912
9	−1	1	1	−1	−1	2352
10	1	−1	−1	−1	−1	1894
11	−1	−1	−1	−1	1	2029
12	1	1	1	−1	−1	1903
13	−1	1	−1	−1	1	1281
14	1	1	−1	1	1	1491
15	−1	1	1	1	−1	274
16	−1	1	1	1	1	3267
17	1	1	1	1	−1	1500
18	1	−1	1	−1	1	1555
19	1	1	1	−1	1	1638
20	1	−1	1	1	1	1766
21	1	−1	−1	1	−1	1985
22	−1	−1	1	1	−1	2379
23	1	−1	1	−1	−1	1262
24	−1	1	1	−1	1	1446
25	−1	1	−1	1	−1	732
26	1	1	−1	−1	−1	750
27	−1	−1	−1	−1	−1	878
28	1	1	−1	1	−1	1345
29	1	1	−1	−1	1	2498
30	−1	−1	1	1	1	2406
31	1	1	1	1	1	2480
32	−1	−1	−1	1	−1	1262

**Table 3:** ANOVA table for 2^5^ factorial experimental design.

Source	Sum of squares	df	Mean square	*F* value	*P* value	
Model	1.442*E* + 007	26	5.547*E* + 005	10.42	0.0080	Significant
*A*—maltose	2.116*E* + 005	1	2.116*E* + 005	3.97	0.1028	
*B*—NaH_2_PO_4_	4.010*E* + 005	1	4.010*E* + 005	7.53	0.0406	
*C*—beef extract	8.096*E* + 005	1	8.096*E* + 005	15.21	0.0114	
*D*—pH	19208.00	1	19208.00	0.36	0.5742	
*E*—moisture	1.312*E* + 006	1	8.327*E* + 005	15.64	0.0108	
Residual	2.662*E* + 005	5	53237.08			
Cor total	1.469*E* + 007	31				

**Table 4:** Independent variables selected for CCD and RSM.

Factors (%)	Symbols	Coded values				
		−**α**	−1	0	+1	+**α**
Moisture	*A*	46.36	60	80	100	113.64
Beef extract	*B*	0.059	0.1	0.2	0.3	0.341
NaH_2_PO_4_	*C*	0.018	0.025	0.0375	0.05	0.0568

**Table 5:** Experimental design and results of the CCD.

Run	Type	Moisture	Beef extract	NaH_2_PO_4_	Response (*Y*) (units/mL)
1	Factorial	−1 (60)	−1 (0.1)	1 (0.05)	2175
2	Center	0 (80)	0 (0.2)	0 (0.0375)	2553
3	Center	0 (80)	0 (0.2)	0 (0.0375)	3150
4	Center	0 (80)	0 (0.2)	0 (0.0375)	2868
5	Factorial	−1 (60)	1 (0.3)	1 (0.05)	2592
6	Factorial	1 (100)	−1 (0.1)	1 (0.05)	2916
7	Axial	1.682 (113.64)	0 (0.2)	0 (0.0375)	1522
8	Axial	0 (80)	0 (0.2)	−1.662 (0.0182)	2731
9	Factorial	1 (100)	1 (0.3)	1 (0.05)	3699
10	Axial	0 (80)	−1.682 (0.34)	0 (0.0375)	2463
11	Center	0 (80)	0 (0.2)	0 (0.0375)	3147
12	Factorial	−1 (60)	−1 (0.1)	−1 (0.025)	1372
13	Factorial	−1 (60)	1 (0.3)	−1 (0.025)	2689
14	Axial	0 (80)	−1.682 (0.06)	0 (0.0375)	3012
15	Factorial	1 (100)	1 (0.3)	−1 (0.025)	2182
16	Factorial	1 (100)	−1 (0.1)	−1 (0.025)	2209
17	Center	0 (80)	0 (0.2)	0 (0.0375)	3048
18	Axial	0 (80)	0 (0.2)	1.682 (0.0568)	3334
19	Center	0 (80)	0 (0.2)	0 (0.0375)	2779
20	Axial	−1.682 (46.36)	0 (0.2)	0 (0.0375)	86

**Table 6:** Results of the regression analysis of the CCD.

Source	Sum of squares	df	Mean square	*F* value	*P* value
Model	1.108*E* + 007	9	1.231*E* + 006	9.76	0.0007
*A*—moisture	1.541*E* + 006	1	1.541*E* + 006	12.21	0.0058
*B*—beef extract	8.531*E* + 005	1	8.531*E* + 005	6.76	0.0265
*C*—NaH_2_PO_4_	1.139*E* + 006	1	1.139*E* + 006	9.03	0.0132
*AB*	1.196*E* + 005	1	1.196*E* + 005	0.95	0.3533
*AC*	2.880*E* + 005	1	2.880*E* + 005	2.28	0.1618
*BC*	1012.50	1	1012.50	8.023*E* − 003	0.9304
*A* ^2^	6.645*E* + 006	1	6.645*E* + 006	52.65	<0.0001
*B* ^2^	234.01	1	234.01	1.854*E* − 004	0.9665
*C* ^2^	1.691*E* + 005	1	1.691*E* + 005	1.34	0.2739
Residual	1.262*E* + 006	10	1.262*E* + 005		
Lack of fit	9.840*E* + 005	5	9.840*E* + 005	3.54	0.0958
Pure error	2.780*E* + 005	5	55596.57		
Cor total	1.234*E* + 007	19			

**Table 7:** Summary of purification of fibrinolytic enzyme from *B. cereus* IND1.

Procedure	Total activity (units)	Total protein (mg)	Specific (units/mg)	Purification (fold)	Yield (%)
Crude enzyme	27300	364	75	1	100
Ammonium sulfate fraction (30%–70% saturation)	16505	128	129	1.72	60.5
DEAE cellulose	5418	23	235	3.1	19.9
Sephadex G-75	3241	11.5	281	3.8	11.8
Casein-agarose	1248	1.3	960	12.8	4.57
